# Acute chloroquine poisoning: A comprehensive experimental toxicology assessment of the role of diazepam

**DOI:** 10.1111/bph.15101

**Published:** 2020-06-18

**Authors:** Dyfrig A. Hughes

**Affiliations:** ^1^ Centre for Health Economics and Medicines Evaluation Bangor University Bangor Wales UK; ^2^ Department of Molecular and Clinical Pharmacology University of Liverpool Liverpool UK

## Abstract

**Background and Purpose:**

Resurgence in the use of chloroquine as a potential treatment for COVID‐19 has seen recent cases of fatal toxicity due to unintentional overdoses. Protocols for the management of poisoning recommend diazepam, although there are uncertainties in its pharmacology and efficacy in this context. The aim was to assess the effects of diazepam in experimental models of chloroquine cardiotoxicity.

**Experimental Approach:**

In vitro experiments involved cardiac tissues isolated from rats and incubated with chloroquine alone or in combination with diazepam. In vivo models of toxicity involved chloroquine administered intravenously to pentobarbitone‐anaesthetised rats and rabbits. Randomised, controlled treatment studies in rats assessed diazepam, clonazepam and Ro5‐4864 administered: (i) prior, (ii) during and (iii) after chloroquine and the effects of diazepam: (iv) at high dose, (v) in urethane‐anaesthetised rats and (vi) co‐administered with adrenaline.

**Key Results:**

Chloroquine decreased the developed tension of left atria, prolonged the effective refractory period of atria, ventricular tissue and right papillary muscles, and caused dose‐dependent impairment of haemodynamic and electrocardiographic parameters. Cardiac arrhythmias indicated impairment of atrioventricular conduction. Studies (i), (ii) and (v) showed no differences between treatments and control. Diazepam increased heart rate in study (iv) and as with clonazepam also prolonged the QTc interval in study (iii). Combined administration of diazepam and adrenaline in study (vi) improved cardiac contractility but caused hypokalaemia.

**Conclusion and Implications:**

Neither diazepam nor other ligands for benzodiazepine binding sites protect against or attenuate chloroquine cardiotoxicity. However, diazepam may augment the effects of positive inotropes in reducing chloroquine cardiotoxicity.

**Linked Articles:**

This article is part of a themed issue on The Pharmacology of COVID‐19. To view the other articles in this section visit http://onlinelibrary.wiley.com/doi/10.1111/bph.v177.21/issuetoc

## Graphical abstract

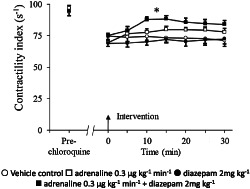

**Abbreviations**
COVID‐19: coronavirus disease 2019
FDA: Food and Drug Administration
LV dP/dt: time derivative of left ventricular pressure
MTBE: methyl‐tertiary‐butyl ether
TSPO: translocator protein

**What is already known**

- Acute chloroquine poisoning manifests as cardiotoxicity and is often managed using diazepam.

**What this study adds**

- Diazepam does not attenuate the effects of chloroquine in isolated cardiac tissues nor in vivo.

**What is the clinical significance**

- Inotropic support, which is essential for chloroquine poisoning, may be potentiated with diazepam.

## 1 INTRODUCTION

Chloroquine and hydroxychloroquine are being repurposed as potential treatments for coronavirus disease 2019 (COVID‐19) (Ferner & Aronson, 2020). The Food and Drug Administration (FDA, 2020) authorised their emergency use in the United States and clinical guidelines in Belgium, China, France, India, Iran, Italy, South Korea and The Netherlands have made recommendations for their use in the prevention and treatment of COVID‐19.

Case reports of cardiotoxicity and fatal poisoning relating to the use of chloroquine and hydroxychloroquine for COVID‐19 have emerged (Agence Régionale de Santé, 2020; Binding, 2020; Busari & Adebayo, 2020; SimpliCity, 2020; Xuan, 2020), as well as excess death with high doses in COVID‐19 clinical trials (Borba et al., 2020). The acute toxic effects of these drugs are well recognised (World Health Organization, 2016) and relate to their cardiotoxic effects of widening of the QRS complex, atrioventricular block, ventricular arrhythmias, negative inotropy, hypotension and severe hypokalaemia, which occur within 1–3 h of ingesting doses >5 g in adults. Without intensive, supportive treatment, circulatory collapse and death can rapidly follow acute overdose. Mortality due to acute toxicity is high, with 134 of the 387 cases reported in the literature between 1955 and 1975 (Bondurand, N'Dri, Coffi, & Saracino, 1980), and a further 135 from 335 suicide attempts (Weniger and World Health Organization, 1979) resulting in death.

Current recommendations for the management of acute toxicity include ensuring adequate ventilation, gastric lavage, administration of activated charcoal, adrenaline for its inotropic and vasoconstrictor effects, diazepam and correction of metabolic acidosis and hypokalaemia (Jones, 2015). The observation in 1976 of a patient who took 5 g of chloroquine together with 500 mg of diazepam and survived without symptoms of chloroquine toxicity (Djelardje, 1976), drew attention to the possible role of diazepam in chloroquine poisoning. Subsequent case reports (Jaeger, Sauder, Kopferschmitt, & Flesch, 1987; Meeran & Jacobs, 1993; Rajah, 1990) and a prospective non‐randomised trial (Riou, Barriot, Rimailho, & Baud, 1988), in which the odds of survival significantly favoured diazepam therapy, led to the recommendation of diazepam in the management of acute chloroquine toxicity. However, there remains controversy given some conflicting evidence of benefit (Demaziere et al., 1992; Clemessy et al., 1996) and limitations in study designs (Yanturali, 2004).

Experimental toxicity studies are also inconclusive. Crouzette, Vicaut, Palombo, Girre, and Fournier (1983) demonstrated that an intraperitoneal injection of diazepam caused a significant decrease in the mortality of rats treated with chloroquine. Riou, Rimailho, Galliot, Bourdon, and Huet (1988) observed an improvement in haemodynamics and a correction of the QRS interval prolongation when diazepam was administered to chloroquine‐intoxicated pigs. Gnassounou and Advenier (1988) observed that clonazepam protected anaesthetised rats against chloroquine toxicity and that diazepam but not the translocator protein (TSPO) agonist Ro5‐4864 (4′‐chlorodiazepam) protected against the decrease in contractions, observed when guinea pig atria were exposed to chloroquine. In other studies, however, diazepam failed to improve the mechanical performance of rat cardiac papillary muscle exposed to chloroquine (Riou, Lecarpentier, Barriot, & Viars, 1989) and was ineffective in reversing chloroquine toxicity in anaesthetised rats (Buckley, Smith, Dosen, & O'Connell, 1996).

It would therefore appear that the effectiveness of diazepam in reversing chloroquine toxicity is equivocal and that the mechanism(s) by which diazepam may exert its effects remain unclear. Due to the resurgence in the use of chloroquine and its structural analogue hydroxychloroquine for COVID‐19, the aim of the present study was to investigate the potential cardioprotective effects of diazepam in experimental models of chloroquine toxicity.

## 2 METHODS

### 2.1 In vitro methods

A series of experiments was conducted to assess the effects of chloroquine and diazepam alone and in combination on the contractility, refractoriness and beating rate of isolated rat cardiac tissues.

#### 2.1.1 Animals

All animal care and experimental procedures were performed in accordance with the UK Animals (Scientific Procedures) Act 1986, approved by the institutional ethical review committee, and conducted under the authority of project licences held at the University of Liverpool. Animal studies are reported in compliance with the ARRIVE guidelines (Kilkenny et al., 2010; McGrath, McLachlan, & Zeller, 2015) and with the recommendations made by the *British Journal of Pharmacology.*

Male Wistar rats were bred in the departmental animal unit (the Nuffield Joint Facilities) or in exceptional circumstances of supply shortage acquired from the Biomedical Services Unit, Faculty of Medicine, or the Department of Veterinary Pathology, University of Liverpool. Rats were kept under conditions of 12‐h light/dark cycle at 20°C with food (CRM diets, SDS, Witham, Essex) and water available ad libitum. The optimal weight range for experimental use was 200–400 g.

#### 2.1.2 Tissue preparation

Rats were administered 1,000 IU·kg^−1^ of sodium heparin by an intraperitoneal injection. After 15 min, they were stunned by a blow to the head, exsanguinated and hearts were excised. Isolated atria, ventricular strips (≤2 mm in width) dissected longitudinally towards the apex of the heart and right papillary muscles were prepared and suspended in 30‐ml organ baths, containing (in mM), NaCl 119; KCI 3.8; MgS0_4_ 1.18; KH_2_PO_4_ 1.18; NaHCO_3_ 25; CaCl_2_ 1.9 plus d‐glucose 10.0, gassed with 95% O_2_, 5% CO_2_ (BOC medical gases, Guildford) and maintained at 37°C. Each preparation was subjected to a resting diastolic tension of 10 mN and stimulated with square wave pulses of 5‐ms duration at a frequency of 1 Hz via a Grass S48 or S88 stimulator (Quincy, Massachusetts). Tissues were stimulated at twice threshold voltage ≤15 V. Right atria were allowed to equilibrate such that spontaneous, rhythmic beating occurred. In all cases, tissues were washed periodically throughout the stabilisation period.

#### 2.1.3 Measurement of cardiac parameters

Contractions were measured isometrically via Dynamometer UF1 transducers (sensitivity range, 559 mN) connected to Lectromed 5230 preamplifiers (Letchworth, Hertfordshire). The beating rate of right atria was measured with a Lectromed 5250 ratemeter preamplifier. These were housed within a MT8P preamplifier unit, which relayed signals to a MT6 thermal pen recorder giving an output on heat‐sensitive paper. Each channel was calibrated such that a full‐scale deflection of 20 mN could be observed. Time to peak tension was measured from the onset of electrical stimulation to the peak of the contraction (Penefsky, 1994). The effective refractory periods (ERPs) of left atria, right papillary muscles and ventricular strips were measured using a modification of the extra stimuli method (Reuter & Heeg, 1971).

#### 2.1.4 Experimental protocol

A target of six samples of each cardiac tissue were assigned at random to one of four concentrations of chloroquine (calculated as the base; 1, 10, 30, or 100 μM, dissolved in Krebs solution) in the presence of the vehicle for diazepam (1% v/v propylene glycol). Spontaneously beating right atria were exposed only to 30‐μM chloroquine. The highest concentration of chloroquine (100 μM) decreased the responsiveness of most tissues after approximately 20 to 30 min. As the threshold voltage for contraction gradually increased, tissues failed to respond to electrical stimuli. It was for this reason that 30 μM was chosen for a subsequent experiment involving diazepam. In this second experiment, fresh tissues (target of six per group) were incubated with diazepam at concentrations of 1, 10 or 100 μM for 30 min before the addition of 30‐μM chloroquine.

### 2.2 In vivo methods

Experimental models of toxicity were developed in spontaneously breathing rats, ventilated rats and ventilated rabbits, in which chloroquine was infused at different rates and measurements taken of haemodynamic and electrocardiographic parameters. Studies were then conducted in which chloroquine‐intoxicated rats were treated with combinations of diazepam, clonazepam, Ro5‐4864, adrenaline or vehicle control.

#### 2.2.1 Experimental protocol

In developing a model of experimental toxicity, animals (six per group) were allocated at random to different doses of chloroquine diphosphate dissolved in 0.9% w/v NaCl. Non‐ventilated rats were randomised to intravenously infused doses (calculated as chloroquine base) of 0.5, 1, 2 or 4 mg·kg^−1^·min^−1^, ventilated rats 1, 2 or 4 mg·kg^−1^·min^−1^and rabbits 0.5, 1,or 2 mg·kg^−1^·min^−1^ for a maximum period of 60 min or until death, after an initial period of stabilisation of at least 20 min.

Six treatment randomised controlled trials were subsequently conducted to assess the efficacy of diazepam, clonazepam and Ro5‐4864:‐ (i) prior, (ii) during and (iii) after chloroquine intoxication (Table 1) and the effects of diazepam:‐ (iv) in high dose, (v) in non‐barbiturate anaesthetised rats and (vi) co‐administered with adrenaline. Six rats were randomised to each treatment group within each of these studies. Benzodiazepines (and vehicles) were administered as a slow intravenous bolus over 2 min.

**TABLE 1:** Experimental protocols for randomised controlled trials (i) to (vi) conducted in anaesthetised rats

Aim	Ventilation	Dose of chloroquine	Treatment timing	Control	Treatment groups
(i) Efficacy of ligands for benzodiazepine binding sites (before infusion of chloroquine)	Spontaneously breathing	2 mg·kg^−1^·min^−1^ until death	10 min before start of chloroquine infusion	1 ml·kg^−1^ of 25% ethanol, 25% polyethylene glycol‐300 in saline (NaCl 0.9%)	1. Diazepam 2 mg·kg^−1^ 2. Clonazepam 1.1 mg·kg^−1^ 3. Ro5‐4864 0.16 mg·kg^−1^
(ii) Efficacy of ligands for benzodiazepine binding sites (during infusion of chloroquine)	Ventilated	1 mg·kg^−1^·min^−1^ for 60 min	30 min following start of chloroquine infusion	1 ml·kg^−1^ of 25% ethanol, 25% polyethylene glycol‐300 in saline (NaCl 0.9%)	1. Diazepam 2 mg·kg^−1^ 2. Clonazepam 1.1 mg·kg^−1^ 3. Ro5‐4864 0.16 mg·kg^−1^
(iii) Efficacy of ligands for benzodiazepine binding sites (after infusion of chloroquine)	Spontaneously breathing	2 mg·kg^−1^·min^−1^ for 15 min	Immediately following cessation of chloroquine infusion	1 ml·kg^−1^ of 25% ethanol, 25% polyethylene glycol‐300 in saline (NaCl 0.9%)	1. Diazepam 2 mg·kg^−1^ 2. Clonazepam 1.1 mg·kg^−1^ 3. Ro5‐4864 0.16 mg·kg^−1^
(iv) Efficacy of high dose diazepam (during infusion of chloroquine)	Ventilated	1 mg·kg^−1^·min^−1^ for 60 min	30 min following start of chloroquine infusion	1 ml·kg^−1^ of 10% N,N‐dimethylacetamide, 5% Tween‐80 in saline (NaCl 0.9%)	1. Diazepam 10 mg·kg^−1^
(v) Efficacy of diazepam (during infusion of chloroquine) with a non‐barbiturate anaesthetic	Ventilated	1 mg·kg^−1^·min^−1^ for 60 min	30 min following start of chloroquine infusion	1 ml·kg^−1^ of 25% ethanol, 25% polyethylene glycol‐300 in saline (NaCl 0.9%)	1. Diazepam 2 mg·kg^−1^
(vi) Efficacy of diazepam and adrenaline (during infusion of chloroquine)	Ventilated	1 mg·kg^−1^·min^−1^ for 60 min	30 min following start of chloroquine infusion	1 ml·kg^−1^ of 25% ethanol, 25% polyethylene glycol‐300 in saline (NaCl 0.9%) (vehicle for diazepam) 0.1 mg·kg^−1^ ascorbic acid 0.01% in saline (NaCl 0.9%) (vehicle for adrenaline)	1. Adrenaline 0.3 μg·kg^−1^·min^−1^ (+ vehicle for diazepam) 2. Diazepam 2 mg·kg^−1^ (+ vehicle for adrenaline) 3. Diazepam 2 mg·kg^−1^ + adrenaline 0.3 μg·kg^−1^·min^−1^

#### 2.2.2 Drug doses

Diazepam was administered in a 2 mg·kg^−1^ intravenous bolus dose, based on Riou, Rimailho, et al. (1988) and 10 mg·kg^−1^ in the study of high dose diazepam, which approximates to the dose recommendations for human cases of overdose (Jones, 2015) when scaled allometrically (Nair & Jacob, 2016). The doses of clonazepam (1.1 mg·kg^−1^) and Ro5‐4864 (0.16 mg·kg^−1^) were chosen to have the equivalent GABAergic and non‐GABAergic activity respectively, to 2 mg·kg^−1^ of diazepam (Wang, Taniguchi, & Spector, 1984). These approximate to human equivalent doses of 0.18 and 0.03 mg·kg^−1^, respectively. The dose of adrenaline (0.3 μg·kg^−1^·min^−1^) was chosen to correspond to that which produced 50% increase in maximum rate of left ventricular pressure (LV + dP/dt_max_) in anaesthetised rats (Latini, Zuanetti, Conforti, Schwartz, & Lazzara, 1988). However, this is appreciably lower (allometrically scaled human equivalent dose of 0.05 μg·kg^−1^·min^−1^) than infusion rates in human poisoning, which are titrated to maintain arterial pressure (median maximal rate of 0.7 μg·kg^−1^·min^−1^) (Mégarbane et al., 2010).

#### 2.2.3 Animals

Male Wistar rats (as above) and female New Zealand White rabbits, which were either bred in the departmental animal unit or purchased from Harlan Interfauna (Huntingdon, Cambridgeshire), were used. Rabbits were housed under ambient conditions of a 12‐h light/dark cycle at 18°C with food (R14 from SDS, Witham, Essex) and given amprolium HCI 7.68% w/v and ethopabate 0.49% w/v (1.5 ml per 500 ml) drinking water for 5 days as a prophylaxis against *coccidiosis* infection. For rabbits, the optimal weight range for experimental use was 2–3 kg.

#### 2.2.4 Anaesthesia

Anaesthesia was induced in rats with sodium pentobarbitone of 60 mg·kg^−1^ intraperitoneally and, once venous access was established, maintained with intravenous boluses of 3 mg as required. In the treatment study (v), urethane was prepared as a 15% w/v solution in isotonic saline and administered as an intraperitoneal dose of 1.4 g·kg^−1^.

Neuroleptanalgesia was induced in rabbits by an intramuscular injection of 0.5 ml·kg^−1^ Hypnorm (0.315 mg·ml^−1^ fentanyl citrate and 10 mg·ml^−1^ fluanisone). Surgical anaesthesia was achieved by administering sequential 4‐mg boluses of sodium pentobarbitone into the marginal ear vein and then 12‐mg boluses via a cannulated femoral vein, as required, upon commencement of ventilation.

#### 2.2.5 Surgical preparation

Femoral veins were cannulated for venous access for drug administration. The right common carotid and a femoral artery were accessed for measurement of left ventricular pressure and recording of BP using a Druck PDCR 75 or a Bell and Howell type 4‐422‐0001 pressure transducer. A tracheotomy was performed to facilitate respiration, and a wide bore cannula secured in place. Subcutaneous stainless‐steel needle electrodes were inserted to each limb for the recording of the ECG. All animals were maintained at a rectal temperature of 37°C.

#### 2.2.6 Mechanical ventilation

Ventilation necessitated a thoracotomy at the fifth intercostal space as, in closed‐chest rats, excessive contractions of the diaphragm and intercostal muscles were found to prevent effective respiration. A positive end‐expiratory pressure was exerted and air ventilation provided at 54 strokes·min^−1^ (3–4.5 ml per stroke) using a Harvard Bioscience small animal respirator. Rabbits were ventilated with air at 38 strokes·min^−1^ (13–18 ml per stroke). A thoracotomy was not necessary in anaesthetised rabbits. Blood gases were measured using a Corning 158 or 850 pH/blood gas analyser. Stroke volumes were adjusted for pre‐drug PO_2_ >80 mmHg and PCO_2_ >30 mmHg.

#### 2.2.7 Exclusion criteria

Animals were excluded with pre‐drug mean arterial BP <60 mmHg (in anaesthetised rats) or <40 mmHg (in ventilated rabbits), arterial PO_2_ of <70 mmHg, arterial PCO_2_ <25 or >40 mmHg or if arrhythmias occurred during the stabilisation phase of the experiment. In the randomised trials, rats were excluded if they died prior to the administration of chloroquine in trial (i) or the treament in trials (ii) to (vi).

#### 2.2.8 Measurement of cardiovascular parameters

Arterial BP, left ventricular pressure, and its first derivative (LV ± dP/dt_max_) and contractility index LV + dP/dt_max_/P, left ventricular end‐diastolic pressure, heart rate and ECG (lead II) were measured and recorded using Lectromed systems (Letchworth, Hertfordshire) or a Grass 79D recorder (Quincy, Massachusetts) connected to a Po‐Ne‐Mah digital data acquisition system (Linton, Diss, Norfolk) and recorded at a sampling rate of 1,000 Hz.

#### 2.2.9 Whole blood concentration of chloroquine

Blood samples for the determination of chloroquine concentration were obtained from trial (iv). Approximately 250‐μl arterial blood samples were drawn after 25, 45 and 60 min of chloroquine infusion from six rats for the analysis of whole blood chloroquine concentrations. Four 50‐μl aliquots were accurately pipetted on to a sheet of Whatman grade 3 blotting paper and protected from light exposure.

Standards were prepared by adding aliquots of chloroquine, giving final concentrations ranging from 0 to 30 μM, on to chloroquine‐free blood spots. All samples were carefully cut from the surrounding paper, macerated and placed in individual glass vials containing 50 μl of a 1 μg·ml^−1^ solution of 7‐chloro‐4‐(5‐diethylamino‐1‐methylpentyl‐amino)‐quinoline diphosphate to serve as an internal standard and 3 ml of 0.2‐M HCI. The vial contents were vortexed, allowed to settle, filtered and added to 0.5 ml of 5‐M NaOH and 2.5 ml each of methyl‐tertiary‐butyl ether (MTBE) and hexane. This was centrifuged and the organic layer separated. Fresh MTBE and hexane were added to the remaining aqueous phase and the extraction procedure repeated. The solvent was evaporated from each organic sample by heating combined with a gentle flow of dry nitrogen. Samples were then stored at −20°C.

Chloroquine was detected using an Isochrom LC Spectra‐Physics pump equipped with a Rheodyne injector, a Spectra 100 fluorescence detector, and a Chromjet integrator. The excitation wavelength was 340 nm, and a 370‐nm emission filter was used (Looareesuwan et al., 1986). The column (0.25 m × 4.6 mm of internal diameter) was packed with Spherisorb silica (5‐μM particles; Capital HPLC) and eluted with an isocratic mobile phase consisting of acetonitrile:methanol:diethylamine (94:5.5:0.5), flowing at 1.5 ml·min^−1^. The limit of detection for chloroquine was 3.1 nM, and the precision of the method was 3.5% at 156 nM.

#### 2.2.10 Biochemical measurements

In randomised trial (vi), arterial blood samples were analysed for blood gases, pH and electrolyte (K^+^, Na^+^, and free Ca^2+^) concentrations using a Corning 850 analyser. Blood samples of approximately 200 μl were collected at baseline, before treatments and 30 min post‐treatment.

#### 2.2.11 Analysis of electrocardiographic parameters

ECG interval measurements were made manually and based on the average of four successive ECG complexes for each recording time point. The QRS interval was measured from the onset of the Q wave (or R wave if no Q was visible) to the point at which the ST segment bisected the isoelectric line. The QT interval was corrected (QTc) using Bazett's (1920) formula.

### 2.3 Drugs and reagents

All salts for Krebs solutions were of AnalaR grade or higher and obtained from BDH, Poole, or Fisons, Loughborough. Chloroquine diphosphate, (±) adrenaline HCl, urethane (ethyl carbamate), Tween 80, N,N‐dimethylacetamide and propylene glycol were obtained from Sigma, Poole. Heparin sodium (Multiparin of 5,000 IU·ml^−1^) injection was obtained from CP Pharmaceuticals, Wrexham. MTBE was purchased from Fisons, Loughborough; Ro5‐4864 was purchased from Fluka Chemika, Gillingham, Dorset; Hypnorm (fentanyl/fluanisone) was from Janssen Animal Health, Petteridge, Kent; Amprol Plus (Amprolium HCl/ethopabate) was from Merk Sharp & Dohme, Hertfordshire; and sodium pentobarbitone was from RMB Animal Health Ltd, Dagenham. 7‐Chloro‐4‐(5‐diethylamino‐1‐methylpentyl‐amino)‐quinoline diphosphate was a gift from the Walter Reed Army Institute of Research, Washington D.C.; and clonazepam and diazepam were gifts from Hoffman‐La Roche, Basel.

### 2.4 Data and statistical analysis

Each experiment involved six independent samples per randomised group. This was not based on any formal calculations, as the minimally important difference and SD of the variables of interest were unknown. Data are presented as means ± SEM of the average of observations over the time course of the experiment. Data were tested for normality using Shapiro–Wilk tests. For multiple (≥3) comparisons, data were compared by one‐way ANOVA followed by a Bonferroni modified *t*‐test where indicated, or by Kruskal–Wallis tests if non‐normally distributed. The post hoc tests were conducted only if the ANOVA *F*‐test achieved *P* < .05 and there was no significant variance inhomogeneity. Comparisons of two groups of data used either an unpaired Student's *t*‐test for normally distributed data or a Mann–Whitney *U* test for skewed data or where there was a significant difference between the variances of each group. Differences between groups in the time to onset of arrhythmias were analysed by log‐rank tests. A *P*‐value less than 0.05 was considered statistically significant. Statistical analyses were performed using Arcus Pro‐Stat version 3.12. The data and statistical analysis comply with the recommendations of the *British Journal of Pharmacology* on experimental design and analysis in pharmacology (Curtis et al., 2018) with the exception that the analysis was not blinded.

### 2.5 Nomenclature of targets and ligands

Key protein targets and ligands in this article are hyperlinked to corresponding entries in http://www.guidetopharmacology.org, the common portal for data from the IUPHAR/BPS Guide to PHARMACOLOGY (Harding et al., 2018), and are permanently archived in the Concise Guide to PHARMACOLOGY 2019/20 (Alexander et al., 2019).

## 3 RESULTS

### 3.1 In vitro cardiac tissues

Tissues from 57 rats (272 ± 4 g) were used. Decreases in the developed tension of left atria were observed with chloroquine at the highest concentration of 100 μM. The negative inotropic effect was time dependent, with maximal changes observed by 30 min. Chloroquine did not significantly alter the developed tension or time to peak tension of right ventricular strips or papillary muscles but significantly increased the time to peak tension in atria (70 ± 4 ms with chloroquine [100 μM] compared to 54 ± 2 ms in the control group). Chloroquine prolonged the ERP of all tissues. In left atria, for instance, the pre‐chloroquine ERP was 41 ± 2 ms (in the 30 μM group), which significantly increased to 72 ± 9 ms after 30‐min exposure to chloroquine.

Diazepam alone was without effect on papillary muscles or ventricular tissue other than a small but significant increase from 65 ± 2 to 74 ± 2 ms in the time to peak tension of contracting right ventricular strips at 100 μM. However, diazepam of 100 μM evoked a positive inotropic response and prolonged the ERP of left atria and had a significant negative chronotropic effect on right atria (213 ± 11 vs. 290 ± 14 beats·min^−1^).

Diazepam in the concentration range of 1 to 100 μM did not appear to protect against the effects of 30‐μM chloroquine (Table 2). At the highest concentration, diazepam lengthened the ERP and extended the time to peak tension in left atria and reduced rate of beating right atria.

**TABLE 2:** Effects of chloroquine (30 μM) in the presence of propylene glycol 1% v/v (control) or diazepam (1, 10 and 100 μM) on the developed tension, effective refractory period and time to peak tension of left atria, right ventricular strips, right papillary muscles and on the spontaneous beating rate of right atria

Tissue		Diazepam		
Measurement	Control	1 μM	10 μM	100 μM
Left atria	*N* = 7	*N* = 6	*N* = 6	*N* = 6
Developed tension (mN)	7.9 ± 0.8	6.3 ± 0.6	7.9 ± 0.7	8.7 ± 0.5
Effective refractory period (ms)	58 ± 5	56 ± 7	71 ± 8	94 ± 10*
Time to peak tension (ms)	44 ± 1	45 ± 1	45 ± 1	51 ± 2*
Right ventricular strips	*N* = 6	*N* = 7	*N* = 8	*N* = 7
Developed tension (mN)	2.3 ± 0.5	2.7 ± 0.6	2.3 ± 0.5	2.4 ± 0.6
Effective refractory period (ms)	131 ± 6	106 ± 10	125 ± 5	146 ± 7
Time to peak tension (ms)	70 ± 2	69 ± 3	70 ± 3	75 ± 3
Right papillary muscles	*N* = 6	*N* = 6	*N* = 6	*N* = 6
Developed tension (mN)	3.0 ± 0.7	2.7 ± 0.7	2.3 ± 0.9	3.3 ± 1.1
Effective refractory period (ms)	112 ± 6	117 ± 7	119 ± 9	147 ± 11
Time to peak tension (ms)	77 ± 1	76 ± 2	77 ± 3	85 ± 5
Right atria	*N* = 5	*N* = 5	*N* = 6	*N* = 6
Beats (min^−1^)	260 ± 17	247 ± 10	273 ± 13	184 ± 9*

*Note*: Data are mean ± SEM.

* *P* < 0.05 versus control group.

### 3.2 In vivo models of experimental toxicity

#### 3.2.1 Haemodynamic effects

**FIGURE 1:**
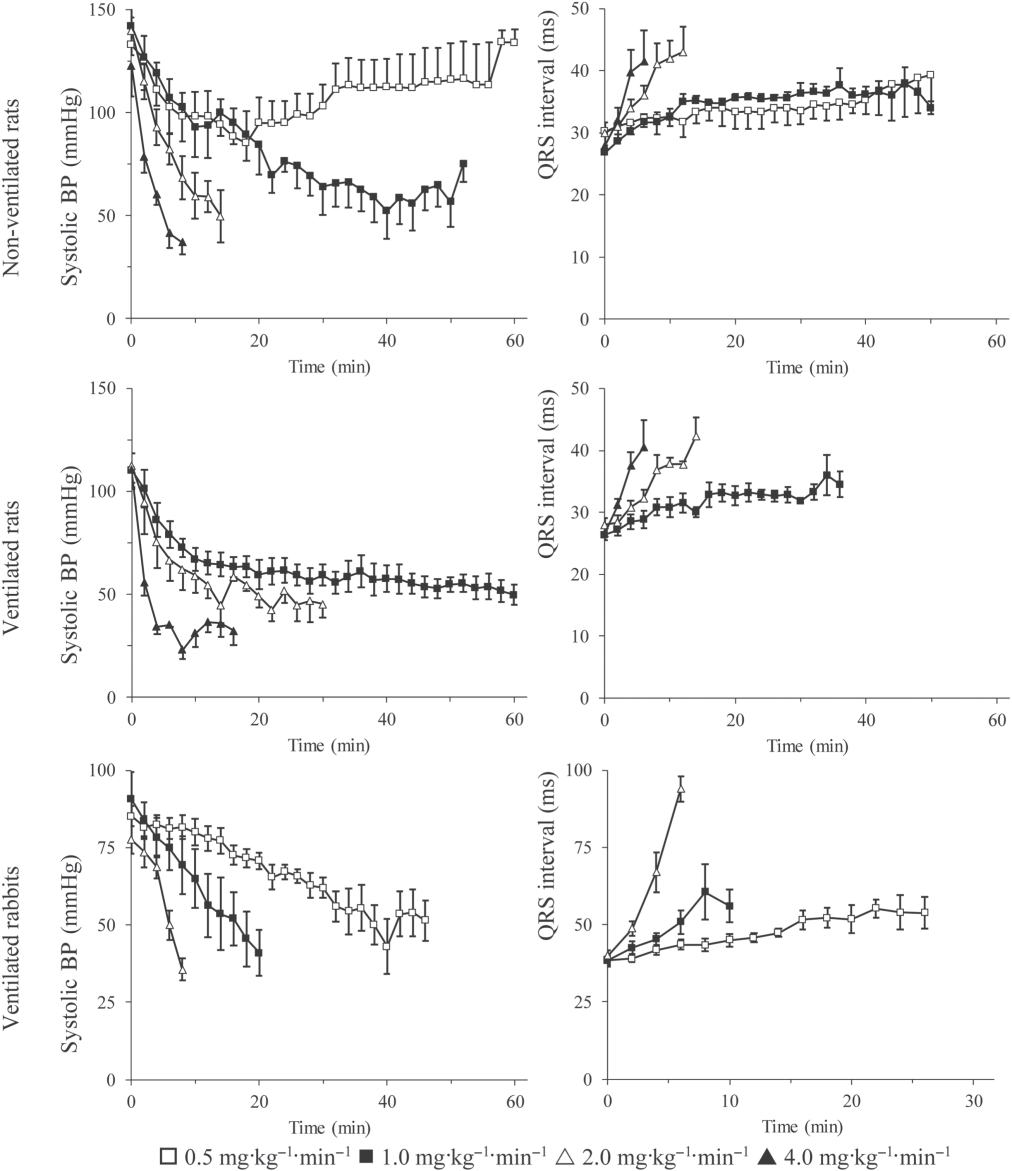
The effects of chloroquine on systolic arterial BP (left panel) and QRS interval (right panel), in spontaneously breathing rats, in ventilated rats, and in ventilated rabbits. Data are expressed as the mean ± SEM

In both rats and rabbits, marked dose‐dependent decreases in systolic and diastolic BP were observed during continuous infusions of chloroquine (Figure 1). In spontaneously breathing rats, the highest dose of chloroquine (4 mg·kg^−1^·min^−1^) caused the most pronounced effect, with a reduction in systolic pressure from 123 ± 15 to 37 ± 6 mmHg occurring during the first 8 min. Similar reductions in pressure were observed in ventilated rats receiving chloroquine although baseline values were lower, as expected in thoracotomized rats. Equivalent depressor responses in rabbits occurred with approximately twofold less chloroquine (on a mg·kg^−1^ basis).

Chloroquine caused dose‐dependent negative inotropy in both species. Reductions in LV + dP/dt_max_ during the first 2 to 4 min of infusion seemed more pronounced than reductions in BP. For example, a 47% reduction in LV + dP/dt_max_ occurred during the first 2 min of infusion at 2 mg·kg^−1^·min^−1^ compared with a 15% reduction in diastolic pressure for the same period in non‐ventilated rats. Cardiac lusitropy (LV − dP/dt_max_) declined in a parallel manner to the negative inotropic response. Increases in left ventricular end‐diastolic pressure were observed with chloroquine in non‐ventilated rats and ventilated rabbits.

Chloroquine caused a similar dose‐dependent bradycardia over the time course of the experiment in both ventilated and non‐ventilated rats for the corresponding doses. In rabbits, however, heart rate declined abruptly by approximately half at time points corresponding to the onset of arrhythmias.

#### 3.2.2 Electrocardiographic effects

In rats, increases in the PR intervals occurred with all doses of chloroquine and in proportion to the cumulative dose received. For example, the PR interval increased from 52 ± 3 to 68 ± 4 ms during the first 12 min in ventilated rats receiving 1 mg·kg^−1^·min^−1^ chloroquine, and from 50 ± 2 to 68 ± 4 ms during the first 6 min at twice the infusion rate, with both groups receiving a total of 12 mg·kg^−1^ of chloroquine over these periods. Chloroquine also caused a dose‐dependent increase in the PR interval in rabbits.

Chloroquine broadened the QRS complex in all animals (Figure 1), although this was not as pronounced with the slower infusion rates as the changes in PR duration. In ventilated rats, for example, the QRS duration increased by 17% in the first 30 min of chloroquine being infused at 1 mg·kg^−1^·min^−1^ while a 30% increase in PR interval occurred over the same period.

QT interval prolongations were observed with high infusion rates, but these were not as apparent when the QT was corrected for rate effects. A substantial increase in QTc occurred only with 2 mg·kg^−1^·min^−1^ in ventilated rats.

Chloroquine induced arrhythmias in 34/38 rats. Typically, impairment of atrioventricular (AV) conduction leading to varying degrees of AV block was associated with ventricular ectopy. Ventricular bigeminy sometimes preceded episodes of ventricular tachycardia in the latter stages of infusion. Ventricular tachycardia was commonly triggered by “R on T” depolarisations. In three rats, the ventricular tachycardia was polymorphic with characteristic features of torsade de pointes. Two rats in each of the ventilated and non‐ventilated groups did not experience cardiac arrhythmias at the lower dose of 0.5 mg·kg^−1^·min^−1^.

In all 16 rabbits, arrhythmias presented as Mobitz type II, second‐degree AV block with a conduction ratio of 2:1 (two P waves for each QRS complex). The onset of arrhythmias was dose dependent and with higher degrees of block eventually occurring at the faster infusion rates. These largely degenerated to ventricular tachycardia and fibrillation.

### 3.3 In vivo drug intervention studies

- Efficacy of ligands for benzodiazepine binding sites (before infusion of chloroquine)

Twenty‐seven rats entered in the study, but three died immediately following the administration of clonazepam and were excluded. There were no differences between pre‐ and 10 min post‐drug haemodynamics or ECG parameters or between randomised groups with the administration of diazepam, clonazepam, Ro5‐4864, or vehicle. In the presence of these agents, chloroquine reduced BP, heart rate, contractility index and increased the PR, QRS and QTc intervals (Table 3). There were no significant differences in these parameters, or in the time to developing cardiac arrhythmias: 18.1 ± 2.8, 15.8 ± 1.6, 17.5 ± 1.2 and 17.8 ± 2.3 min, between the control, diazepam, clonazepam and Ro5‐4864 groups, respectively.

- Efficacy of ligands for benzodiazepine binding sites (during infusion of chloroquine)

Twenty‐five rats entered the study, but one was excluded having died before being administered the treatment drug/vehicle. Following 30 min of chloroquine infusion, there were similar, significant changes in haemodynamics and ECG intervals across drug/vehicle treatment groups (Table 3). There were no differences in any of the measured parameters between intervention and groups following the administration of treatment. However, all rats survived without developing arrhythmias.

- Efficacy of ligands for benzodiazepine binding sites (after infusion of chloroquine)

Three rats were excluded as they died prior to the end of chloroquine infusion. A further four rats died before the end of the experiment but were included in the study, one each from the clonazepam and control groups and two from the Ro5‐4864 group. There was an imbalance in pre‐chloroquine baseline QRS intervals, with higher values in those randomised to Ro5‐4864 compared with other groups. Over 15 min, chloroquine caused significant changes in all cardiovascular parameters in all randomised groups. However, there was a difference between groups in the QTc interval, which was shorter in the control versus diazepam, clonazepam or Ro5‐4864 groups. Following the cessation of chloroquine administration and administration of randomised treatment, all parameters rapidly returned to baseline values in all groups (Table 3). Heart rate was higher and QTc interval prolonged in both the diazepam and clonazepam groups compared with control. There was no significant difference in the incidence of cardiac arrhythmias between drug treatment/vehicle groups.

- Efficacy of high dose diazepam (during infusion of chloroquine)

In contrast to trial (ii), an infusion of chloroquine for 30 min did not cause significant changes in any of the haemodynamic or ECG parameters. Chloroquine did not reduce mean BP and heart rate or increase the QTc interval significantly in those randomised to diazepam and did not increase the QRS interval in either group. Following treatment, heart rate increased significantly in the diazepam group. There were no differences between treatment groups in other parameters and none developed arrhythmias (Table 3).

**TABLE 3:** Impact of drug treatmnents on haemodynamic and electrocardiographic parameters in chloroquine intoxicated, anaesthetised rats (*n* = 6 per group)

Intervention	Mean BP (mmHg)	Heart rate (min^−1^)	Left ventricular +dP/dtmax/P +dP/dtmax†	PR interval (ms)	QRS interval (ms)	QTc interval (ms)	Arrhythmias (incidence)
(i) Administered before chloroquine infusion							
Control	69 ± 6	331 ± 20	84 ± 5	57 ± 3	36 ± 2	191 ± 11	4/6
Diazepam	63 ± 4	327 ± 17	85 ± 4	67 ± 3	37 ± 1	185 ± 10	5/6
Clonazepam	70 ± 6	323 ± 11	85 ± 2	63 ± 2	36 ± 2	182 ± 5	5/6
Ro5‐4864	61 ± 3	307 ± 7	76 ± 3	61 ± 3	36 ± 2	191 ± 9	5/6
(ii) Administered during chloroquine infusion							
Control	57 ± 5	278 ± 11	68 ± 3	69 ± 3	34 ± 1	182 ± 4	0/6
Diazepam	58 ± 4	301 ± 11	72 ± 3	70 ± 2	34 ± 1	193 ± 3	0/6
Clonazepam	58 ± 5	280 ± 16	70 ± 3	76 ± 2	37 ± 2	199 ± 8	0/6
Ro5‐4864	63 ± 8	280 ± 16	77 ± 3	76 ± 7	33 ± 1	189 ± 4	0/6
(iii) Administered after chloroquine infusion							
Control	79 ± 7	314 ± 9	89 ± 4	53 ± 3	31 ± 1	156 ± 4	1/6
Diazepam	97 ± 7	348 ± 12*	89 ± 6	56 ± 2	31 ± 0	173 ± 2*	0/6
Clonazepam	85 ± 8	356 ± 5*	91 ± 4	59 ± 3	31 ± 1	172 ± 2*	1/6
Ro5‐4864	82 ± 8	336 ± 16	87 ± 4	53 ± 2	31 ± 1	160 ± 3	2/6
(iv) High dose diazepam administered during chloroquine infusion							
Control	86 ± 6	311 ± 7	88 ± 3	61 ± 1	31 ± 1	193 ± 2	0/6
Diazepam	82 ± 5	367 ± 9*	90 ± 4	57 ± 4	32 ± 2	203 ± 7	0/6
(v) Administered during chloroquine infusion with urethane anaesthetic							
Control	46 ± 2	318 ± 12	3,202 ± 750^a^	70 ± 1	35 ± 1	203 ± 4	0/6
Diazepam	48 ± 2	345 ± 15	2,728 ± 206^a^	65 ± 2	34 ± 1	198 ± 3	0/6
(vi) Administered during chloroquine infusion							
Control	47 ± 5	258 ± 10	74 ± 3	84 ± 6	35 ± 1	212 ± 4	1/7
Adrenaline	60 ± 2	291 ± 13	78 ± 8	73 ± 2	31 ± 0*	205 ± 5	0/6
Diazepam	46 ± 6	278 ± 18	70 ± 3	76 ± 4	34 ± 2	204 ± 5	1/7
Diazepam + adrenaline	54 ± 3	290 ± 15	84 ± 3*	70 ± 4	34 ± 1	215 ± 4	1/7

*Note*: Data are mean ± SEM, of time‐averaged measurements taken over 15 min during administration of chloroquine in trial (i), and over 30 min following administration of drug trestments in trials (ii) to (vi).

a [+dP/dtmax/P] was measured in each experiment with the exception of experiment (v), which measured [+dP/dtmax].

* *P* < 0.05 versus control group.

The whole blood chloroquine concentration in these rats was 12.2 ± 0.8 μM after 25 min of infusion (1 mg·kg^−1^·min^−1^), 14.3 ± 0.9 μM after 45 min and 16.3 ± 1.1 μM after 60 min.

- Efficacy of diazepam (during infusion of chloroquine) with a non‐barbiturate anaesthetic (urethane)

Over 30 min of administration, chloroquine only significantly affected the PR and QRS intervals. There were no subsequent differences between groups, following administration of diazepam or vehicle control, in any of the measured parameters and none developed arrhythmias (Table 3). A further randomised trial was initiated with chloroquine infused at a higher rate of 2 mg·kg^−1^·min^−1^ in order to evaluate the effects of diazepam on more pronounced toxicity. However five of the first nine rats died and the study was terminated.

- Efficacy of diazepam and adrenaline (during infusion of chloroquine)

**FIGURE 2:**
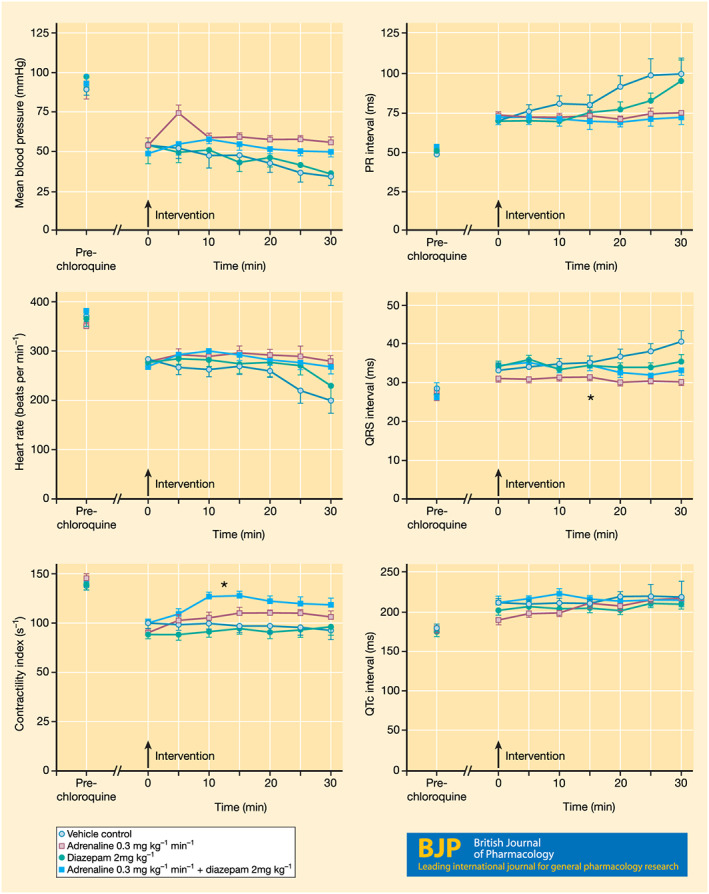
The effects of diazepam, adrenaline, alone and in combination, administered as an treatment at 30 min during a 60‐min infusion of chloroquine (1 mg·kg^−1^·min^−1^) on mean BP, heart rate, contractility index, PR interval, QRS interval, and QTc interval. Data are expressed as the mean ± SEM. Timings are relative to the administration of the drug/vehicle treatment. *P < 0.05 versus other groups

Twenty‐seven rats was included, but three died before the end of the experiment, one from each of the control, diazepam and diazepam + adrenaline groups. During the first 30 min of infusion, chloroquine caused significant changes in all parameters, with the exception of the QRS and QTc intervals in the adrenaline group. The lack of an effect with diazepam (2 mg·kg^−1^) alone was consistent with trial (ii). The effects of adrenaline alone did not deviate significantly from the control group in any parameter other than the QRS interval, but this was not prolonged following chloroquine (Figure 2). The combined administration of diazepam and adrenaline resulted in an improvement of cardiac contractility compared to the control and diazepam groups but not the adrenaline group (84 ± 3 vs. 78 ± 8 s^−1^). No significant differences were observed in the other parameters or incidence of arrhythmias (Table 3).

Pre‐chloroquine potassium concentrations were in the range expected for rats (Burns & De Lannoy, 1966). Chloroquine alone did not cause any significant changes in arterial PO_2_, PCO_2_ or pH values over a period of 30‐min infusion. The combined administration of diazepam and adrenaline, however reduced PO_2_ when compared to pre‐treatment values but not when compared to the other groups. Chloroquine did not alter electrolyte concentrations; but pre‐treatment groups containing adrenaline were more hypokalaemic than the diazepam and control groups (Table 4).

**TABLE 4:** Whole blood pH, gas, and electrolyte concentrations measured at baseline (pre‐chloroquine), following 30 min of chloroquine infusion (pre‐drug treatment/vehicle), and 30 min post‐treatments

	Control	Adrenaline	Diazepam	Diazepam + adrenaline
Baseline (pre‐chloroquine)				
PO_2_ (mmHg)	83 ± 3	77 ± 3	83 ± 3	84 ± 2
PCO_2_ (mmHg)	25 ± 2	24 ± 1	28 ± 1	29 ± 2
pH	7.42 ± 0.02	7.45 ± 0.01	7.45 ± 0.02	7.44 ± 0.02
[K^+^] (mmol·L^−1^)	2.18 ± 0.32	2.34 ± 0.24	2.41 ± 0.29	2.21 ± 0.40
[Na^+^] (mmol·L^−1^)	143.8 ± 2.5	142.2 ± 1.0	142.1 ± 1.5	142.9 ± 0.9
[Ca^2+^] (mmol·L^−1^)	0.82 ± 0.07	0.89 ± 0.05	0.91 ± 0.07	0.89 ± 0.10
Pre‐drug/vehicle treatment				
PO_2_ (mmHg)	79 ± 4	78 ± 3	76 ± 4	80 ± 6
PCO_2_ (mmHg)	22 ± 1	23 ± 2	24 ± 2	27 ± 3
pH	7.42 ± 0.01	7.43 ± 0.02	7.38 ± 0.02	7.41 ± 0.03
[K^+^] (mmol·L^−1^)	1.90 ± 0.25	2.05 ± 0.31	2.01 ± 0.23	2.35 ± 0.40
[Na^+^] (mmol·L^−1^)	145.9 ± 1.7	143.5 ± 1.7	144.9 ± 2.1	144.3 ± 1.1
[Ca^2+^] (mmol·L^−1^)	0.72 ± 0.07	0.79 ± 0.06	0.76 ± 0.05	0.84 ± 0.08
30 min post‐ drug/vehicle				
PO_2_ (mmHg)	74 ± 3	67 ± 5	76 ± 5	65 ± 3*
PCO_2_ (mmHg)	26 ± 2	29 ± 3	27 ± 2	32 ± 2
pH	7.23 ± 0.04*	7.31 ± 0.03*	7.31 ± 0.04	7.26 ± 0.02*
[K^+^] (mmol·L^−1^)	2.21 ± 0.26	1.71 ± 0.18*	2.73 ± 0.16	1.52 ± 0.22*
[Na^+^] (mmol·L^−1^)	147.3 ± 1.8	144.8 ± 2.2	143.6 ± 1.5	148.1 ± 1.8
[Ca^2+^] (mmol·L^−1^)	0.78 ± 0.05	0.81 ± 0.05	0.84 ± 0.04	0.77 ± 0.06

*Note*: Data are mean ± SEM.

* *P* < 0.05 versus pre‐drug/vehicle treatment (within‐group).

## 4 DISCUSSION

The results of the study indicate that chloroquine prolongs the ERP of isolated rat atria. In vivo experiments also revealed that chloroquine induces cardiac arrhythmias and pathophysiologic changes in haemodynamic and electrocardiographic parameters. Different protocols of diazepam administration did not result in significant improvement in cardiac function either in vitro or in vivo. However, the administration of diazepam and adrenaline in combination may be effective against chloroquine cardiotoxicity by improving cardiac contractility.

The findings from the in vitro studies are congruent with previous experiments demonstrating the acute cardiotoxic effects of chloroquine (Essien & Ette, 1986; Tona, Ng, Akera, & Brody, 1990). At the concentrations of chloroquine used, left atria were more sensitive to detrimental effects on mechanical performance than either ventricular or papillary tissue preparations. Decreases in developed isometric tension, together with increases in times to peak tension, were observed, which are indicative of impaired atrial contractility. An increase in the time to peak tension by chloroquine reflects a prolongation of one or more phases of the cardiac excitation–contraction cycle and is consistent with the ability of chloroquine to block cardiac ion channels (Essien & Ette, 1986; Rodríguez‐Menchaca et al., 2008; Sánchez‐Chapula, Salinas‐Stefanon, Torres‐Jácome, Benavides‐Haro, & Navarro‐Polanco, 2001; Tona et al., 1990). Ikhinmwin, Sofola, and Elebute (1981) demonstrated a negative inotropic response which was reversed in the presence of increased extracellular calcium aimed to promote calcium influx via unblocked L‐type calcium channels. Tona et al. (1990) demonstrated chloroquine to inhibit the Treppe response in atrial guinea pig preparations, but without effect on post‐extrasystolic potentiation of contractile force, suggesting that chloroquine interferes with cellular calcium influx upon which the Treppe response is dependent, but not the latter response which is dependent on intracellular calcium mobilisation. Increases in the refractoriness of cardiac tissues are indicative of potassium and/or sodium ion channel blockade. Using voltage‐clamped cat ventricular myocytes, Sánchez‐Chapula et al. (2001) observed that chloroquine blocked several inward and outward membrane currents. The order of potency (1–10 μM range) was inward rectifying potassium current > rapid delayed rectifying potassium current > sodium current > L‐type calcium current. Neither the transient outward potassium current nor the slow delayed rectifying potassium current was modified by chloroquine. Salinas and Cebada (1993) also demonstrated that chloroquine blocks the inward rectifying potassium current in dog cardiac myocytes but had no effects on either the transient outward or the delayed rectifier currents. Rodríguez‐Menchaca et al. (2008) established that chloroquine blocks the inward rectifier K_ir_2.1 channels, that underlie the cardiac inward rectifier potassium current, from the cytoplasmic surface. Other quinolone antimalarials also have known actions in modulating cardiac electrical activity, including blockade of human ether‐a‐go‐go related gene (hERG) potassium and L‐type calcium channels (Coker, Batey, Lightbown, Díaz, & Eisner, 2000; Kim, Lee, Cha, Kwon, & Kim, 2010; Michel, Wegener, & Nawrath, 2002).

Diazepam had little effect on the function of myocardial tissue, other than at 100 μM, where it increased the ERP and peak developed tension in left atrial preparations and increased the times to peak tension in right ventricular strips. Diazepam inhibits PDE4 (Collado et al., 1998), suggesting a possible mechanism for cardioprotection, although this occurs at lower concentrations (IC_50_ of 8.7 μM) than required to elicit responses in the present investigation. The responses of cardiac tissues to chloroquine in the presence of diazepam were no different from vehicle controls, supporting previous observations that diazepam does not attenuate the cardiac effects of chloroquine via a direct action upon the heart (Riou et al., 1989).

The in vivo experimental models of chloroquine toxicity indicated that impaired cardiac contractility was the primary event in the sequalae of toxicity. Hypotension, bradycardia, changes in ECG intervals, arrhythmias and death followed in a similar manner as described previously (Sofola, 1980). However, cardiac arrhythmias may be less prevalent in cases of human chloroquine poisoning which occurs following oral ingestion (absorption half‐life ~20 min) and where blood concentrations are predominantly governed by the distribution and redistribution processes from the various body compartments back to the intravascular space (Mégarbane et al., 2010). Differences between species in cardiac electrophysiology may also explain varying arrhythmic manifestations. The provision of mechanical ventilation did not appear to influence the onset or the severity of these effects. Significant changes in cardiovascular function occurred in the absence of changes in either arterial blood gas levels or pH, suggesting that toxic manifestations due to chloroquine are not secondary to hypoxia. Chloroquine was about twice as potent in its toxic effects in rabbits than in rats (on a mg·kg^−1^ basis), where whole blood concentrations were within the 10–20 μM range. This concentration range is comparable with the concentrations used in the in vitro experiments and observed in human toxicokinetic studies. Clemessy et al. (1995) reported a mean whole blood chloroquine concentration of 20.1 μM (range 1.8 to 78 μM) among 191 patients admitted to intensive care. Mégarbane et al. (2010) correlated mild cardiotoxicity with peak concentrations ≤16 μM, moderate 16–25 μM and severe >25 μM. Chloroquine is extensively distributed to extravascular tissues and some reversal of cardiotoxicity would be expected upon cessation of administration. The in vivo experimental models of chloroquine toxicity did not test this. However, in experiment (iii), the administration of chloroquine (2 mg·kg^−1^·min^−1^ i.v.) ceased after 15 min and recovery in cardiovascular parameters was observed in all treatment/vehicle groups.

The series of randomised controlled trials was designed to assess whether modulation of the GABA_A_ receptor or other effects of diazepam might account for previous reports of reduced toxicity with chloroquine. However, diazepam, whether administered prior, during or after the administration of chloroquine or at high dose, failed to attenuate chloroquine‐induced cardiotoxicity in anaesthetised rats. These results are consistent with previous studies in spontaneously breathing rats anaesthetised with thiobutobarbitone (Buckley et al., 1996), but contrast with experiments performed in conscious rats (Crouzette et al., 1983) and pentobarbitone‐anesthetised, mechanically ventilated pigs (Riou, Rimailho, et al., 1988). Possible explanations for these discrepancies might include the choice of species, doses of chloroquine and diazepam, and anaesthesia. Based on allometric scaling to human doses, 2 and 10 mg·kg^−1^ of diazepam administered to rats correspond to 0.3 and 1.6 mg·kg^−1^ in adults. Effects were similar in the trial in which urethane was chosen as an anaesthetic for its lack of interaction with GABA_A_ receptors.

Experiments aimed to differentiate any GABA mediated versus other effects of diazepam, used Ro5‐4864, which has activity at the mitochondrial TSPO benzodiazepine binding site distinct from the GABA_A_ receptors in the CNS and clonazepam, which rapidly crosses the blood–brain barrier and is a potent positive allosteric modulator of GABA_A_ receptors, while having low affinity towards TSPO. Mitochondrial TSPO is ubiquitously expressed in various tissues, including the heart with a putative role in regulating heart rate and contractility (Surinkaew, Chattipakorn, & Chattipakorn, 2011). As neither diazepam nor either of these agents protected against or attenuated chloroquine toxicity, it is unlikely that any cardiovascular effects in the context of chloroquine toxicity can be attributed to interaction with benzodiazepine binding sites.

In view of the fact that the principal adverse effect of chloroquine is negative inotropy (Sofola, 1980) and the absence of positive inotropic effects of diazepam under basal conditions (and negative inotropy under certain conditions [Zeegers, van Wilgenburg, & Leeuwin, 1998]), the use of a positive inotrope seems essential for the improvement in the cardiac function following chloroquine toxicity. While neither diazepam nor adrenaline alone reversed any chloroquine‐induced cardiovascular changes, the improvement in cardiac contractility observed with their combined administration may indicate a beneficial interaction. Studies in rat ventricular tissues demonstrated that diazepam (10 μM) augmented contractility due to isoprenaline (Martinez, Peñafiel, Collado, & Hernández, 1995), noradrenaline (Juan‐Fita, Vargas, & Hernández, 2003) and dopamine (Juan‐Fita, Vargas, & Hernández, 2006). These effects were not mimicked by GABA nor antagonised by the selective TSPO inhibitor PK11195 or flumazenil, an antagonist of the GABA_A_ benzodiazepine binding site. Rather, they were attributed to diazepam's ability to inhibit PDE4, the main isoenzyme responsible for the inotropic effect of β‐adrenoceptor agonists in the rat myocardium. This offers a plausible mechanism for the observed effects in chloroquine‐intoxicated rats. However, there are differences between species in the expression of PDE4, with a fivefold higher amount of non‐PDE4 activity in human hearts compared to rodents, and this will impact on the effect of enzyme inhibition (Richter et al., 2011). Further mechanistic studies are warranted to assess the role of PDE4 inhibition in this context.

In conclusion, the results of this study do not offer compelling support for the use of diazepam in reducing chloroquine cardiotoxicity. Ligands for benzodiazepine binding site, clonazepam, and Ro5‐4864 were similarly ineffective in the experimental models used. However, the results provide evidence that diazepam might enhance cardiac contractility when co‐administered with adrenaline, although the lowering of whole blood potassium concentrations, consistent with agonism of β_2_‐adrenoceptors in skeletal muscle, might risk exacerbation of chloroquine‐induced hypokalaemia (Clemessy et al., 1995) and increased arrhythmogenicity.

These new insights have important clinical and research implications in the current context of widespread publicity and use of chloroquine for COVID‐19. Chloroquine is widely used and available without prescription in many countries, including the United Kingdom, presenting dangerous opportunities for unintentional overdose. For the management of patients with chloroquine poisoning, testing the efficacy of a positive inotrope with a greater selectivity for β_1_‐adrenoceptors, such as dobutamine, would be desirable, as would a greater understanding of the non‐cardiovascular roles for diazepam treatment given that chloroquine poisoning often causes convulsions, which can be intractable.

## AUTHOR CONTRIBUTIONS

D.A.H. designed the study, performed all experimental work, analysed the data, and drafted the manuscript. D.A.H. approves the version to be published and agrees to be accountable for all aspects of the work in ensuring that questions related to the accuracy or integrity of any part of the work are appropriately investigated and resolved.

## CONFLICT OF INTEREST

The author declares no conflicts of interest.

## DECLARATION OF TRANSPARENCY AND SCIENTIFIC RIGOUR

This Declaration acknowledges that this paper adheres to the principles for transparent reporting and scientific rigour of preclinical research as stated in the *BJP* guidelines for Design and Analysis and Animal Experimentation, and as recommended by funding agencies, publishers, and other organisations engaged with supporting research.
